# Quantitative Estimation of Aflatoxin Level in Poultry Feed in Selected Poultry Farms

**DOI:** 10.1155/2022/5397561

**Published:** 2022-01-31

**Authors:** Muhammad Naveed, Kashif Syed Haleem, Shakira Ghazanfar, Isfahan Tauseef, Naseem Bano, Charles Oluwaseun Adetunji, Muhammad Hamzah Saleem, Huda Alshaya, Bilal Ahamad Paray

**Affiliations:** ^1^Department of Microbiology, Hazara University Mansehra, 21300, Pakistan; ^2^Functional Genomics and Bioinformatics, National Agricultural Research Centre, Islamabad 45500, Pakistan; ^3^National Institute for Genomics Advanced Biotechnology, National Agricultural Research Centre, Park Road, Islamabad-45500, Pakistan; ^4^Livestock Research and Development Station, Peshawar, Pakistan; ^5^Applied Microbiology, Biotechnology and Nanotechnology Laboratory, Department of Microbiology, Edo State University Uzairue, Edo State, Nigeria; ^6^College of Plant Science and Technology, Huazhong Agricultural University, Wuhan 430070, China; ^7^Cell and Molecular Biology, University of Arkansas, Fayetteville 72701, USA; ^8^Department of Zoology, College of Science, King Saud University, P.O. Box 2455, Riyadh 11451, Saudi Arabia

## Abstract

*Statement of Novelty*. Poultry feed contamination due to mycotoxins is one of the major threats to the growing poultry industry. Surveillance of different mycotoxins, including aflatoxin, is very important to control economic and health hazards associated with these toxins. Studies reporting aflatoxin levels in poultry feed are limited. Therefore, this study was conducted to examine the occurrence of total aflatoxin in poultry feed. This study is the first-ever documentation about the frequency and quantitative estimations of total aflatoxin levels in poultry feed consumed to provide solid feedback to the poultry industrialists and researchers involved in studying the mycotoxins. *Objective*. Contamination of poultry feed with mycotoxins such as aflatoxin is a major concern for the poultry industry that results in a significant economic loss and directly affects consumers. Monitoring the aflatoxin levels in poultry feed is crucial for controlling economic loss and decreasing the health hazards to the population. This study was conducted to examine the occurrence of total aflatoxin in poultry feed in a high consumption area. Three different poultry feeds, i.e., starter, grower, and finisher, were assessed through continuous sampling from farms. The incidence of positive samples for aflatoxin contamination was 92.5%. Grower feed had the highest frequency (100%) of aflatoxin positive samples and aflatoxin levels with a mean value of 56.34 ppb. Further, the range of moisture content was around 6.8%-10.98%. No significant correlation between humidity and aflatoxin contamination was revealed when analyzed by Pearson's correlation coefficient with *r*^2^ of 0.05 and *p* value of 0.13. The results warrant the need for constant monitoring programs for the prevention of aflatoxin contamination in local poultry farms

## 1. Introduction

*Aspergillus* is soil fungal species that have been recognized as a major contaminant of different grains utilized for poultry diets [1]. They grow rapidly under high moisture conditions and produce biologically active hepatotoxic aflatoxins [2]. Maize, cereals like rice, wheat, pistachios, cottonseed, copra groundnuts, and many other feed stuffs are contaminated by these fungal species [3–5]. Aflatoxin actually covers three words in accordance with its definition, i.e., “a” from genus *Aspergillus*, “fla” from *flavis* species, and toxin means poison [6]. Aflatoxins are the most studied toxins due to their association with high morbidity and mortality rate in poultry. Therefore, aflatoxins in several feed grains are a major public health concern, which adversely affects human and animal health [5]. Among livestock and poultry, this toxin is mainly responsible for aflatoxicosis, and the disease leads to acute suppression of the immune system and a low production rate [7]. Several other studies have reported that aflatoxin has carcinogenic and hepatotoxigenic effects as well [8–10]. It has the ability to cross the placenta and cause slow growth rate of neonates [11, 12]. Toxins such as aflatoxin B1 affects lungs, causing different diseases in animals [10, 13]. Contamination of food or feed of poultry with fungal toxins reduces its safety and quality causing a huge loss at the industrial level. However, Food and Drug Administrative Authority considers aflatoxin as an unavoidable food contaminant and has set regulatory levels for it. The permissible level for poultry is 20 ppb [14, 15]. The toxin production can take place in either preharvest or postharvest stage of the crop. In addition to feed, aflatoxin can also be found in dairy milk products of animals exposed to these feeds. The environmental factors directly effecting the aflatoxin production include temperature, oxygen, and moisture contents. Usually, the production of aflatoxin G occurs at 28°C while that of aflatoxin B typically takes place at 11-37°C [16]. As the temperature in many areas increases due to sudden global changes, a higher temperature can facilitate the production of fungi, e.g., *Aspergillus* species that flourish in warmer climatic regions. This, in turn, results in increased aflatoxin production [17]. Pakistan is one of the countries affected adversely due to climate change. Therefore, an increased risk of aflatoxin is expected in the coming few years. In Pakistan, the poultry industry contributes 26.8% to total meat production. The poultry sector has emerged as a source of employment for more than 1.5 million people in the last few years [18]. Poultry feeds/ingredients have been routinely polluted by aflatoxins in different regions of Pakistan [19–21]. Different studies have reported different ratios of aflatoxin B1 and B2 contamination in the feed of poultry starter chick and poultry starter broiler with concentrations ranging between 100 and 320 *μ*gkg^−1^ [19]. Keeping in view the importance of aflatoxins, the present study was conducted to investigate the occurrence of total aflatoxin in poultry feed consumed in poultry farms located in Northern Pakistan. The selected area has a high consumption of poultry feed due to an increased number of farms but lacks any authentic data about the frequency and levels of aflatoxin contamination. This study is the first-ever documentation about the frequency and quantitative estimations of total aflatoxin level in poultry feed consumed in Northern Pakistan in order to provide a solid feedback to the researchers involved in studying the mycotoxins.

## 2. Material and Methods

The study was performed on broiler poultry feed supplied in different poultry farms of Northern Pakistan through a random feed sampling method. A framework was designed with the help of previous research done in the field of aflatoxin.

### 2.1. Sample Collection

Forty samples of poultry feed were collected from local poultry farms of Northern Pakistan in 4 months (March 2018 to July 2018). The feed samples represented three poultry feed categories: broiler-starter feed (1-19 days), broiler-grower feed (19-30 days), and broiler-finisher feed (31-50 days). The collected samples were ground, carefully mixed, and then stored at 2-8°C (35-46°F) in sealed plastic bags.

### 2.2. Sample Extraction

For extraction, 70% methanol solution was prepared by mixing seven parts of ACS grade methanol with three parts of distilled water. For every tested sample, the sample was ground and 5 grams of it was vigorously shaken in 25 mL of methanol for four minutes. 5 ml of the suspension was filtered through a Whatman filter, and the filtrate was collected for further assays.

### 2.3. Detection of Aflatoxin through Direct ELISA

The level of aflatoxin was determined through ELISA using the kit and protocol supplied by Neogen Veratox Corporation USA. Briefly, 100 *μ*l conjugate was added to red marked mixing wells to initiate the process of aflatoxin detection. A 100 *μ*l of each standard (0 ppb, 5 ppb, 15 ppb, and 50 ppb) was added in the first four red microwells. Similarly, 100 *μ*l of sample extract was added in the 5^th^ red microwell. The conjugate and sample extract in all red wells were thoroughly mixed by a multichannel pipette, and 100 *μ*l from each red microwell was transferred to the corresponding white microwells. The solution in white microwells was gently shaken for 10 to 15 seconds and incubated for two minutes. Following this, the liquid from white wells was removed and the wells were thoroughly washed five times with deionized water. The wells were dried on an absorbent paper towel, and 100 *μ*l of the substrate provided within the kit was added in each white microwell and gently shaken (10 to 15 times). After three minutes of incubation, 100 *μ*l of red stop solution was added into white microwells. The results were obtained on an ELISA reader at 650 nm within 20 minutes of solution addition.

### 2.4. Calculation of the Moisture Content

Hot air oven method was used to determine the dry factor following the standard procedures. The samples were weighed before drying and again after drying at 105°C.

The moisture content was measured using the following equation:

(1) $$\begin{array}{c} \text{Dry factor}\%=\frac{W2-W1}{\text{weight of sample}}\times 100, \end{array}$$

(2) $$\begin{array}{c} \text{Moisture}\%=100-\text{dry factor}, \end{array}$$

where *W*2 is the weight after drying and *W*1 is the weight before or empty crucible dish.

## 3. Results

### 3.1. Frequency of Aflatoxin in Feed Samples in All Samples Collected from Selected Regions of Northern Pakistan

**Figure 1:**
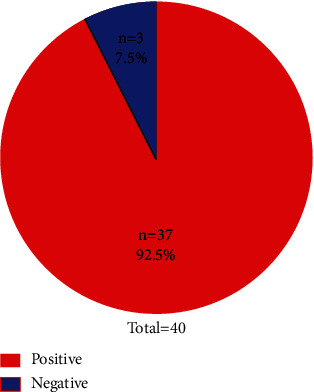
Frequency of positive and negative samples.

A total of 40 feed samples were analyzed from selected regions of Northern Pakistan. Among all 40 tested samples, 92.5% (*n* = 37) were found positive for aflatoxin, whereas only 7.5% (*n* = 3) were negative (Figure 1). Hence, a high percentage of samples contaminated with aflatoxins was evident.

### 3.2. Regions-Wise Frequency, Total Aflatoxin Level, and Moisture Content in Poultry Feed Supplied to Northern Pakistan

**Figure 2:**
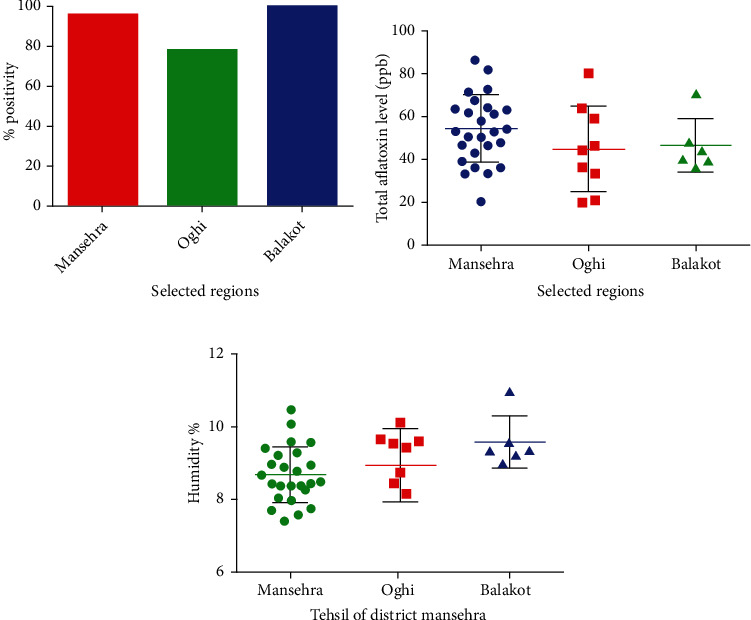
(a) Frequency, (b) total aflatoxin level, and (c) moisture content in poultry feed in selected regions of Mansehra, Pakistan. Results shown in (a) are represented in percentages, whereas results in (b, c) are shown as mean ± SEM of parts per billion and percent humidity of 40 samples tested.

The frequency of aflatoxin was also determined in selected regions (Mansehra, Oghi, and Balakot) of Northern Pakistan. The highest frequency of aflatoxin was reported in samples obtained from the Balakot region where 100% of feed samples were contaminated with aflatoxins. The samples obtained from the Mansehra region showed 96.0% of contamination, while those collected from the Oghi region had 80% aflatoxin contamination (Figure 2(a)).

Mean aflatoxin level in parts per billion was highest (54.56 ppb) in the Mansehra region, followed by the Balakot region (46.5 ppb) and Oghi region (44.8 ppb). There was no significant difference when mean parts per billion values were compared between each region (Figure 2(b)). The highest percentage of moisture content was detected in Balakot (9.56%), followed by Oghi (9.43%) and Mansehra (8.41%). Mean values of Balakot region were significantly higher when compared with Mansehra (with a *p* value of 0.015 by Student's unpaired *t*-test), whereas the rest of the comparisons did not reveal any statistical difference (Figure 2(c)).

### 3.3. Frequency, Total Aflatoxin Levels, and Moisture Contents in Different Types of Poultry Feed

**Figure 3:**
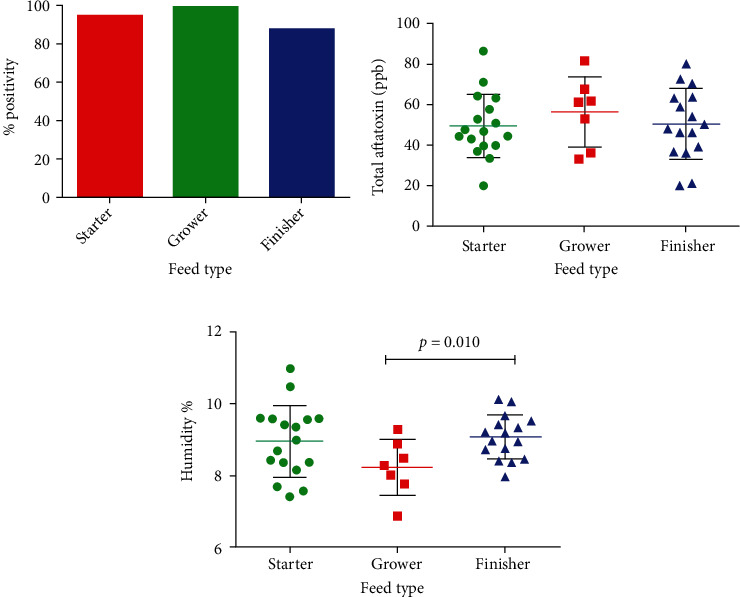
(a) Frequency, (b) total aflatoxin level, and (c) moisture content in different feed types. Results shown in (a) are represented in percentages, whereas results in (b, c) are shown as mean ± SEM of parts per billion and percent humidity of 40 samples tested.

Three different types of feed, i.e., broiler-starter feed, broiler-grower feed, and broiler-finisher feed, were targeted for a detailed evaluation of aflatoxin contamination. The highest frequency of aflatoxin positive samples was detected in grower feed samples (100%) followed by starter feed samples (94.4%) and in finisher (87.5%) (Figure 3(a)). Mean aflatoxin level in parts per billion was highest in grower feed samples (56.34 ppb) followed by finisher feed (50.38 ppb) and starter (49.52 ppb). There was no significant difference when mean parts per billion values of different feed types were compared (Figure 3(b)). However, the mean moisture content level was highest in finisher feed samples (9.07%) followed by starter (8.95%) and grower feed (8.22%). Mean values of moisture content in the finisher feed were significantly higher when compared to the grower with *p* value of 0.01 by Student's unpaired *t*-test. In contrast, the rest of the comparisons did not reveal any statistical difference (Figure 3(c)).

### 3.4. Total Aflatoxin Level and Moisture Contents in Poultry Feed of Different Feed Companies

**Figure 4:**
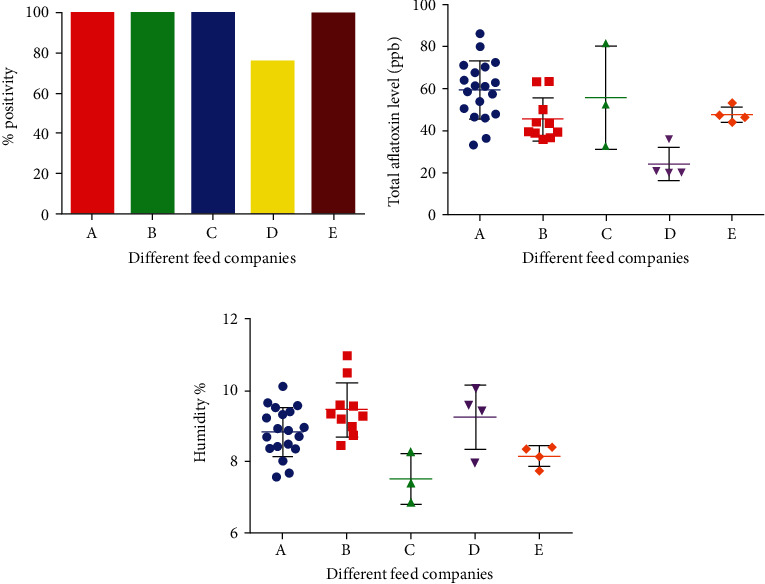
(a) Frequency, (b) total aflatoxin level, and (c) moisture content in poultry feed of different companies. Results shown in (a) are represented in percentages, whereas results in (b, c) are shown as mean ± SEM of parts per billion and percent humidity of 40 samples tested.

All 40 samples investigated in this study were from five different feed companies designated as A, B, C, D, and E. Names of companies are not mentioned to adhere to ethical guidelines. Feed from all the companies tested had 100% aflatoxin, except company D which had 75% positive aflatoxin samples (Figure 4(a)). Mean parts per billion of aflatoxin varied among different companies with the highest values reported from company A (59.50 ppb), followed by company C (59.50 ppb), E (47.8 ppb), B (45.6 ppb), and D (24.27 ppb) (Figure 4(b)). However, the highest moisture content was reported in company B (9.46%), followed by D (9.26%), A (8.84%), E (8.15%), and C (7.41%) (Figure 4(c)).

### 3.5. Correlation between Total Aflatoxin Level and Moisture Content

**Figure 5:**
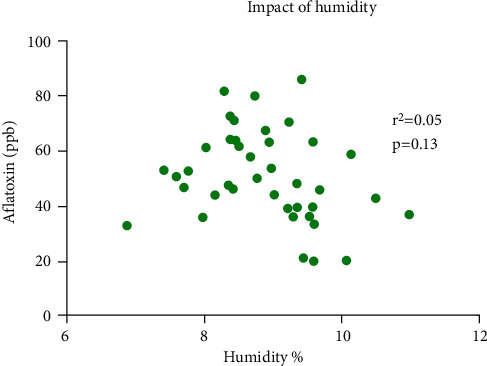
Correlation between moisture contents and total aflatoxin level of poultry feed using Pearson's correlation coefficient. Each dot represents one sample, whereas *Y* axis shows respective aflatoxin level in parts per billion, and *X* axis represents respective level of humidity in percent.

Correlation between moisture content and aflatoxin level was evaluated by drawing a scattered graph. Pearson's correlation coefficient revealed no significant degree of correlation between these two parameters. *r*^2^ was 0.05 with *p* value of 0.13 (Figure 5).

## 4. Discussion

Aflatoxins are toxic fungal metabolites that naturally contaminate poultry feed. *A. flavus* and *A. parasiticus* are the major species that produce these toxic secondary metabolites [22]. High temperature and humidity are optimal for mold growth and toxin production [23, 24]. According to the present study results, 92.5% of samples were found positive for the detection of aflatoxin ranging between 34 and 86.2 ppb. Such a high percentage of total aflatoxins might be due to the lack of awareness for proper storage of chicken feedstuff at the farm. In addition, other factors such as the lack of systems for appropriate airing and maintaining a suitable temperature are also likely to play a role in a higher percentage of contamination. Globally, different studies have reported varying frequencies and levels of aflatoxin. Rossi et al. [25] reported aflatoxins in 88.2% of samples collected from Brazil and analyzed through indirect competitive ELISA. Our findings are in good agreement with this study. Similarly, Anjum et al. [19] documented the high levels of AFB1 in commercial poultry feed ingredients collected from a specific region of Pakistan. Zinedine et al. [26] collected a few poultry feed samples (*n* = 21) from Morocco and observed that the percentage of contamination by aflatoxin is about 66.6%. Dawlatana et al. [27] reported the frequency and the level of aflatoxin in poultry feed from India and Bangladesh. In Bangladesh, the level ranged from 7 to 160 ppb, while in India, 60% of positive samples contained >10 ppb aflatoxins. Ata-Ur-Rehman et al. [28] reported that 42% of commercial poultry feed was contaminated with aflatoxins, which was quite low as compared to the findings of our study. The present study revealed that the level of total aflatoxin is higher in poultry feed of the Mansehra region, which is following the findings of Cavalheiro [29] who reported that aflatoxins are recurrent contaminants of harvested feed and foods and other grains stored under high-temperature conditions. Although moister content levels of the Mansehra region are lower compared to Oghi and Balakot regions, the temperature range of the Mansehra region is quite higher as compared to the other two, which may be the reason for higher aflatoxin contamination in feeds stored in this region. The level of total aflatoxin in poultry grower feed was found high compared to the other two feeds, i.e., starter and finisher. In different feed ingredients, the high levels of aflatoxin affect the quality of the grower feed. According to Xiulan et al. [30], the higher level of aflatoxin in grower feed is due to its ingredients. These findings are also in agreement with the results of del Pilar Monge et al. [31] who analyzed 108 poultry feed samples from Argentina and reported that 79 percent of grower poultry feed samples exceeded the maximum acceptable total fungal count which is an indication that aflatoxin levels may be higher due to high fungal counts. According to Ewaidah [32], 38% of grower feed samples were higher than the FDA plan of 20 *μ*gkg^1^. In contrast, Anjum et al. [19] reported high levels of aflatoxins in chick starter and broiler ranging from 100 to 320 *μ*gkg^−1^ among 700 samples tested. In conclusion, the present study provides information about the occurrence of aflatoxin in the broiler feed of District Mansehra Pakistan. From 40 samples investigated, about 92.5% were found positive in total aflatoxin. Three samples (7.5%) had quantities of 20 ppb in total aflatoxin. These figures are very high as compared to many of the studies reported previously. The results warrant the need for surveillance and constant monitoring programs. Considerably higher concentrations of total aflatoxin might risk the poultry industry with increased economic losses. To ensure food safety, aflatoxin contamination in different types of poultry feed should be monitored.

## Data Availability

All data generated and analyzed during this study are included in this published article.
